# Test of the Practicality and Feasibility of EDoF-Empowered Image Sensors for Long-Range Biometrics

**DOI:** 10.3390/s16121994

**Published:** 2016-11-25

**Authors:** Sheng-Hsun Hsieh, Yung-Hui Li, Chung-Hao Tien

**Affiliations:** 1 Department of Photonics, National Chiao Tung University, 1001 University Road, Hsinchu 30010, Taiwan; jack10313.eo00g@nctu.edu.tw (S.-H.H.); chtien@mail.nctu.edu.tw (C.-H.T.); 2 Department of Computer Science & Information Engineering, National Central University, No. 300 Zhongda Road, Zhongli District, Taoyuan 32001, Taiwan

**Keywords:** wavefront coding, extended depth of field, iris recognition, biometrics

## Abstract

For many practical applications of image sensors, how to extend the depth-of-field (DoF) is an important research topic; if successfully implemented, it could be beneficial in various applications, from photography to biometrics. In this work, we want to examine the feasibility and practicability of a well-known “extended DoF” (EDoF) technique, or “wavefront coding,” by building real-time long-range iris recognition and performing large-scale iris recognition. The key to the success of long-range iris recognition includes long DoF and image quality invariance toward various object distance, which is strict and harsh enough to test the practicality and feasibility of EDoF-empowered image sensors. Besides image sensor modification, we also explored the possibility of varying enrollment/testing pairs. With 512 iris images from 32 Asian people as the database, 400-mm focal length and F/6.3 optics over 3 m working distance, our results prove that a sophisticated coding design scheme plus homogeneous enrollment/testing setups can effectively overcome the blurring caused by phase modulation and omit Wiener-based restoration. In our experiments, which are based on 3328 iris images in total, the EDoF factor can achieve a result 3.71 times better than the original system without a loss of recognition accuracy.

## 1. Introduction

Biometric recognition has been applied to many practical uses, including homeland security, e-commerce or other authentication management purposes. Basically, the personal attributes used for authentication were classified into two parts: (1) physiological attributes, such as DNA, facial features, retinal vasculature, fingerprint, hand geometry, iris texture and so on; and (2) individual behavior features, such as signature, keystroke, voice, and gait style [1]. Among these features, iris texture is one of the most attractive modalities because of its inherent distinctiveness, high stability over time and low risk of circumvention [2]. 

**Figure 1:**
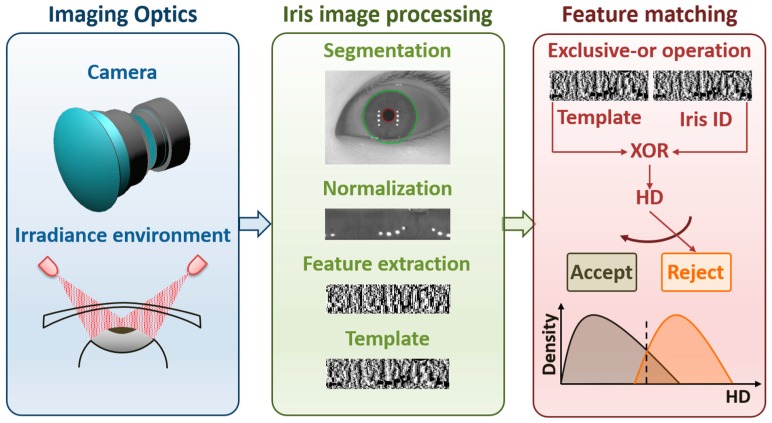
An iris recognition system is composed of the imaging optics unit, the iris image processing unit and the feature matching unit, respectively.

An iris recognition system consists of modules of the imaging optics unit, the image processing unit and the feature matching unit, as shown in Figure 1. The optical system, involving the camera and the irradiance, is used to capture a distant iris image with the highest fidelity possible. The captured images are subsequently processed through many steps. Firstly, the iris images are segmented by determining the centers and radii of the pupillary and limbic boundaries. A conventional segmentation method, such as an integro-differential operator [2,3,4] or Hough transform [4,5], can be applied. Then the iris images are normalized by transforming the coordinates from Cartesian to Polar accordingly. The prominent features of the iris texture are extracted using Gabor filters. Finally, the features are thresholded into binary codes (called iris codes) for the recognition algorithm [2,3,4]. Matching two iris codes using the bit-wise XOR operation generates a distance score. The distance score, Hamming Distance (HD), is employed to measure the distance between two iris codes. An appropriate threshold value of HD is determined so that a decision of acceptance or rejection can be made. For example, two iris images are said to be independent if their HD is above a certain threshold, which is about 0.33 according to Daugman’s algorithm [6]. Otherwise they are assumed to be a match.

For the practical scenario in iris recognition, the acquisition volume, which is defined as the depth of field (DoF), should be large enough to preserve the high reliability and robustness of the system. Imaging optics with sufficient DoF while preserving satisfactory spatial resolution is highly desirable. The conventional approach to increase the DoF is to increase the F-number, which corresponds to using a smaller aperture or longer focal length. However, both scenarios have a side effect. A smaller aperture would lead to a poor optical throughput, and thus a low signal-to-noise ratio; a longer focal length would reduce the field-of-view (FoV), thereby adversely affecting the resolution of the system. Computational imaging proposed by Dowski and Cathey engineered the pupil function to resolve this dilemma in a successful way. After that, many studies applied the coded image for iris recognition and extended the acquisition volume without loss of recognition accuracy [7,8,9,10,11]. To our understanding, numerous previous studies were addressed by the simulation, where the phase mask is assumed to be on the pupil plane exactly. However, for most practical uses, the pupil plane is unreachable by end users because it is hidden inside a complex optomechanical layout. Meanwhile, imperfect irradiances such as glare reflection, non-uniform distribution and brightness level should be considered as well. 

In this paper, we implemented the wavefront coded iris recognition system starting from the acquisition optics to the final score-matching stage. We experimentally compared the recognition performance with different enrollment/testing schemes. The results offer some insight for utilizing the wavefront coding image to provide maximal allowable DoF while maintaining the high recognition accuracy. 

The remainder of this paper is organized as follows. Section 2 introduces the optical consideration and the corresponding terminologies. In Section 3, an extended depth of field (EDoF) system for iris recognition is implemented [12]. In Section 4, the experimental results are examined in terms of various figure of merits, including equal error rate (EER) and HD distributions. In Section 5, discussions on homogeneous or heterogeneous iris recognition are carried out to explore the performance difference between different setups. Section 6 concludes the paper.

## 2. Optical Consideration

### 2.1. Tradeoff between Resolution and Field-of-View

The major challenge of a prime lens for iris recognition lies in a constant acquisition volume (which can be expressed as resolution × FoV). The iris images need sufficient sampling resolution to ensure recognition performance. At the same time, the FoV should be wide enough to cover the entire ocular region and localize the facial landmarks. ISO/IEC 19794-6 suggests that the sampling rate across the iris region should exceed at least 150 pixels so as to contain sufficient features [13]. For an image sensor with a pixel size d, the minimum width of iris images $D_{1}^{\prime }$ is given by:

(1) $$D_{1}^{\prime }=150\times d,$$

With average width of an adult’s iris $D_{1}$ = 12 mm [14], the magnification m of the camera can be obtained as:

(2) $$m=\frac{D_{1}^{\prime }}{D_{1}}\geq \frac{150\times d}{12},$$

The effective focal length f of a camera is related to the magnification *m* and object distance $S_{o}$ [15]:

(3) $$f=\frac{m}{1+m}S_{o},$$

**Figure 2:**
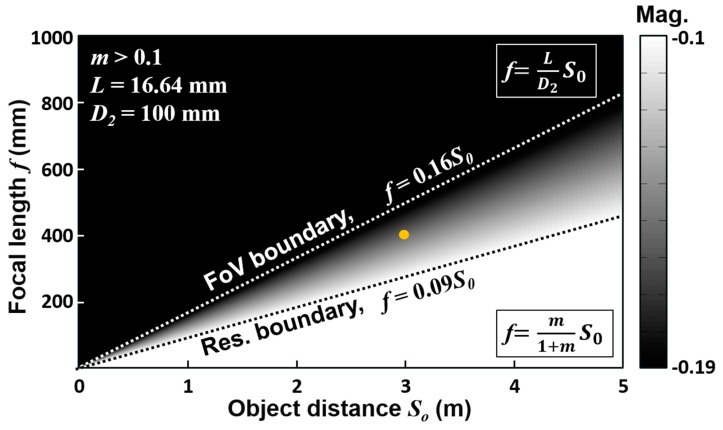
The focal length *f* is constrained by two boundaries for the resolution and the field of view. For object distance *S_o_* = 3 m, the available focal length is in range of *f* = 272–499 mm. In this study, the focal length is set to 400 mm and illustrated as the orange point in this figure.

For the sensor pixel size d = 8 μm in our case, by Equations (2) and (3), the magnification m ≥ 0.1 and focal length $f\geq 0.09\;S_{o}$, which defines the lower bound in terms of resolution. As shown in Figure 2, in case of $S_{o}$ = 3 m working distance, the available focal length should be larger than 272 mm.

On the other hand, another boundary of focal length was defined by the FoV, which is given by:

(4) $$\text{FoV}=2tan^{-1}\left(\frac{D_{2}}{2S_{0}}\right)=2tan^{-1}\left(\frac{L}{2S_{i}}\right),$$

where $D_{2}$ is the width of full ocular region. The ratio of $D_{2}/S_{o}$ in object space is equivalent to the $L/S_{i}$ is image space, where L is the full size of an image sensor and $S_{i}$ is the image distance. For the distant imaging, the paraxial approximation holds that $S_{i}\sim f$, Equation (4) can be rewritten as:

(5) $$f=\frac{L}{D_{2}}S_{o},$$

Since the FoV should be large enough to encompass the entire ocular region, with typical size of an adult’s ocular region $D_{2}$ = 100 mm and the available sensor diameter L = 16.64 mm, Equation (5) defines the upper boundary, $f=0.16S_{o}$, as shown in Figure 2. For the object distance $S_{o}$ = 3 m, the available focal length should be smaller than 499 mm accordingly. Taking both resolution and FoV into account, we employed a commercial telephoto lens, Sigma APO, with 400-mm effective focal length. Detailed specifications of the image sensor and lens set are listed in Table 1.

### 2.2. Depth of Field

**Figure 3:**
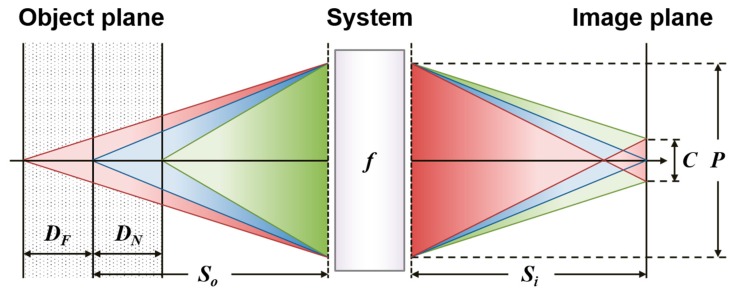
Schematic illustration of the DoF (dotted zone) in an imaging system. DoF is determined by four factors: circle of confusion *C*, exit pupil *P*, object distance *S_o_* and focal length *f*, respectively.

Figure 3 illustrates the concept of DoF, which is marked as dotted zone on the left. When the subject is out of DoF, the point spread functions (PSFs) of the imaging system would increase by the path-length error. The most common merit to evaluate the defocus extent is the circle of confusion (CoC), which is defined as the largest blur PSFs indistinguishable from two distant point sources. For computational imaging with the aid of post-processing, currently there is no universal definition for CoC in optics. In our work, we defined CoC as the maximally allowable iris blurring with acceptable recognition performance [7]. Under the paraxial approximation, the imaging condition at the near and far limits of the DoF ($D_{N}$ and $D_{F}$) can be described as:

(6) $$\frac{1}{S_{i}}+\frac{1}{S_{o}}=\frac{1}{f},$$

(7) $$\frac{1}{S_{i}\left(1+\frac{C}{P-C}\right)}+\frac{1}{S_{o}-D_{N}}=\frac{1}{f},$$

(8) $$\frac{1}{S_{i}\left(1+\frac{C}{P+C}\right)}+\frac{1}{S_{o}+D_{F}}=\frac{1}{f},$$

where P and C are the diameter of the pupil and CoC, respectively. According to Equations (6)–(8), the DoF of an imaging system can be obtained as:

(9) $$D_{N}=\frac{CS_{o}\left(S_{o}-f\right)}{fP+C\left(S_{o}-f\right)},$$

(10) $$D_{F}=\frac{CS_{o}\left(S_{o}-f\right)}{fP-C\left(S_{o}-f\right)},$$

(11) $$DoF=D_{N}+D_{F}=\frac{2CS_{o}}{fP/\left(S_{o}-f\right)-C^{2}\left(S_{o}-f\right)/fP},$$

In our case with P = 86 mm, f = 400 mm and C = 0.136 mm, the DoF is merely 60 mm, which is too shallow to be operated in a robust way. Motion blur inevitably occurs if users are allowed to move or walk, like the use case reported in [12]. One scheme is to increase the shutter speed or F-number by sacrificing the image brightness, which degrades the image quality with a low signal-to-noise ratio. In this study, we resort to the computational image, which cleverly enlarges the acquisition volume without any possible thermal hazard or glare reflection by strong irradiance. 

### 2.3. Irradiation Condition

The performance of an iris recognition system depends greatly on captured image quality. Without cooperation of the subject, image quality is subject to many factors like the low contrast, the inconsistent illumination or the specular reflection. The low contrast is due to the reason that human iris has lower reflectance under visible light but higher reflectance under near infrared (NIR) light [16]. To overcome this issue, we equipped two LED illuminators (BE-IR80L, BlueEyes Technology, TW), which have 850 nm central wavelength and 50 nm full width at half maximum (FWHM). The average irradiance in continuous mode is about 13 mW/cm^2^ to acquire enough information. We set the ISO value of the camera so that the iris image is dark when the NIR LEDs were in off state. Therefore, no NIR pass filter is needed. To take the specular reflection into consideration, we set the incident angle from the illuminators to 35 degrees. When the subject is at 70 cm from the illuminators, such geometry can avoid strong specular reflection even when the subjects wear glasses. An adequate irradiance setup can enhance the probability of the correctness of iris segmentation [17].

## 3. Method to Extend the Depth-of-Field

As the preceding discussion (Section 2.2) states, an iris image with insufficient DoF would inevitably cause motion blur and reduce recognition accuracy. Although decreasing the aperture size is the easiest way to alleviate the phase degradation which is quadratically proportional to the pupil size, smaller apertures will be accompanied by insufficient irradiance and low signal-to-noise ratio. In order to overcome this issue, we applied computational imaging techniques to extend DoF. Computational imaging integrating optics with post signal processing can keep the PSF more robust toward defocusing. Two issues are addressed about the coding scheme. One is to find the appropriate function of the phase mask on the basis of merit. The other is about the coding strength of the phase mask. In addition to coding strategy, decoding is a counterpart issue about the performance of recognition. The intermediate iris image with blurriness undergoes an approximately linear transformation, which ideally can be reversed by a linear reconstruction. A matched filter thus can fully restore the original iris texture. However, noise prevents the reconstruction from being perfect in practical case. In the second part of this section, we examine the decoding scheme coupled with matching algorithm. The following issues are addressed in this section: function of phase modulation with respect to optical transfer function (OTF) analysis; optimal coding strength, which is a tradeoff between increasing defocus insensitivity and loss of information; and the restoration process with optimal matched filter design parameters.

### 3.1. Wavefront Coding

Generally, there are two strategic approaches for phase modulation. One is to use a free-form phase plate, whose distribution is expressed as a polynomial expansion [18]:

(12) $$\varphi \left(x,y\right)=\sum_{n=0}^{k}\left(\sum_{m=0}^{n}C_{nm}x^{n}y^{n-m}\right),$$

where $C_{nm}$ are a set of coefficients that will be determined by the optimization algorithm to balance the factor of EDoF and zero nulls over a broad band range of DoF. Such free-form phase masks have circular symmetric OTFs. The major challenge for the free-form phase plate lies in its fabrication tolerance. More than 10 dominant coefficients in shape formulation would result in difficulties with fabrication [19]. Meanwhile, tilt or alignment error would drastically reduce the performance in an unexpected way. In contrast, the more popular scheme in phase coding is to use a separable function like the cubic phase form P(x,y) in the rectangle coordinate [20]:

(13) $$P\left(x,y\right)=exp\left[i\alpha \left(x^{3}+y^{3}\right)\right],$$

where *x* and *y* are the normalized pupil coordinates. The phase coding strength, α, is determined by the numerical evaluation. In our study, we chose cubic phase form to be our candidate because the mask is easier to be fabricated and implemented in an iris recognition system.

We utilized the optical software Zemax^TM^ to compromise the coding strength of the cubic phase mask and a quadratic defocus term $W_{02}\left(x^{2}+y^{2}\right)$ [20]. Unlike the conventional approach which finds the coding strength based on diffraction-limited OTF in simulation, we set the PSF similarity as the merit function and find its mean-square-error (MSE) through the focus range. With different coding strengths, the PSF similarity and its derivative with respect to defocus provide insight into the optical layout that we could conduct in optical design. It should be noticed that the OTF of the coded system cannot cross zero, because the null point in the OTF will lead to permanent loss of information which cannot be restored by post-processing [20,21,22]. The worse situation is that when the strong coding is imposed, the negative value (contrast reversal) occurred. In order to keep the system within a safe margin, we allow OTF threshold at the Nyquist frequency to be larger than 0.169, which ensures most information is above the noise floor and thus well recoverable [23,24]. With an off-the-shelf telephoto lens system with focal length *f* = 400 mm (F/6.3), the maximum value of α is 42, which enables a three-fold DoF. The feasibility of EDoF was convincingly demonstrated in our simulation which coincides with the prior literatures [25,26,27]. 

### 3.2. Restoration Decoding Process

The coded PSFs are restored by the Wiener filter, which is one of the best known approaches to linear image restoration [28,29]. The Wiener filter expressed in Fourier domain (*u*, *v* are spatial frequencies in *x* and *y* direction, respectively) can be formulated as:

(14) $$\hat{F}\left(u,v\right)=\left[\frac{1}{H\left(u,v\right)}\frac{{\left|H\left(u,v\right)\right|}^{2}}{{\left|H\left(u,v\right)\right|}^{2}+S_{\eta }\left(u,v\right)/S_{f}\left(u,v\right)}\right]G\left(u,v\right),$$

where H(u,v) is the coded transfer function, G(u,v) is the intermediate iris image. The ratio $S_{\eta }\left(u,v\right)/S_{f}\left(u,v\right)$ is the noise-to-signal ratio (NSR) of the imaging system, where $S_{\eta }\left(u,v\right)$ and $S_{f}\left(u,v\right)$ is the power spectrum of the noise and the ideal image, respectively. Generally, the NSR of an imaging system is unknown, and it can only be obtained empirically. We conducted a preliminary test to fine-tune the NSR parameter (denoted as *R*) used in Wiener filtering. 

**Figure 4:**
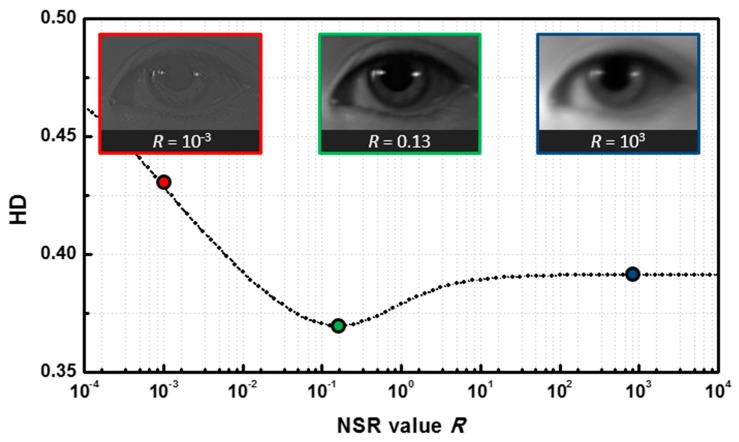
Hamming distance (HD) with different parametric estimation *R* in the Wiener filter, where the HD are averaged based on 64 intra-class comparisons. The optimal *R* is about 0.15.

Iris images of 64 subjects are collected and inversely filtered by Wiener filtering with different *R* values. Then those iris images are used as probe images to match with the iris images in gallery (iris images captured at on-focus position). In principle, Wiener filtering should not affect the inter-class iris matching scores since they are intrinsically different. For the purpose of decreasing computational complexity, we only consider the intra-class comparisons. By accumulating all HD of intra-class comparisons, we obtained the relation between the averaged HD and parameter *R*, as shown in Figure 4. Since HD indicates the distance of two iris images, by locating the minimum of the curve, we are able to estimate the optimal parameter *R* that leads to the best inverse filtering performance. The optimal *R* is found near 0.15 in our case. 

## 4. Laboratory Experimentation

### 4.1. Optical Quality

**Figure 5:**
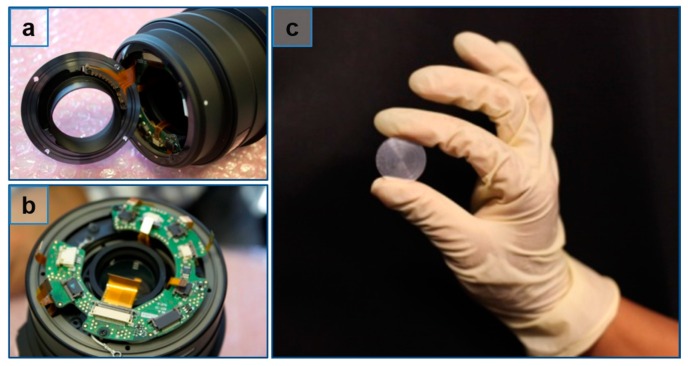
Cubic phase mask with optimized coding strength α = 42 was imbedded into the off-the-shelf telephoto camera, where the mask was placed at the rear space of the system. (**a**) The off-the shelf telephoto lens; (**b**) the mask holder; (**c**) the cubic phase mask.

**Figure 6:**
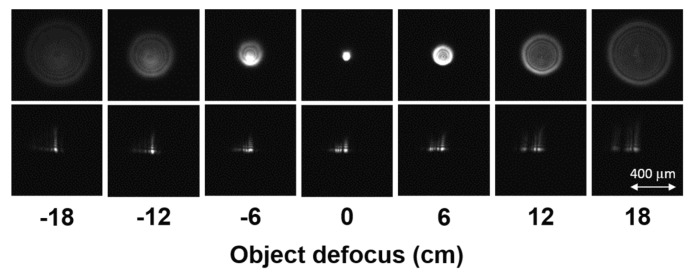
PSFs with different defocus position for top row: conventional, and bottom row: EDoF. Compared with conventional optics whose PSFs are quadratically broadened by defocus, EDoF enables PSFs to be more robust against the defocus.

We embedded a cubic phase mask with optimized coding strength *α* = 42 into the off-the-shelf telephoto camera, as shown in Figure 5, where the phase mask was fabricated by the diamond turning process. We implemented wavefront coding by putting the cubic phase mask at the rear space of the system. The influence of mask displacement away from the focus has been examined by a series of testing in our past research work. Interested readers can refer to our previous research [30]. Figure 6 shows the PSFs across a range of object distances from −18 to 18 cm. It is apparent that the cubic mask helps to reduce the spreading of PSFs. When the FWHM of PSF is comparable to the size of CoC, the object defocus corresponds to −30 mm and +40 mm, which is very close to the theoretical prediction of 60 mm in Section 2.2. 

### 4.2. Image Quality

**Figure 7:**
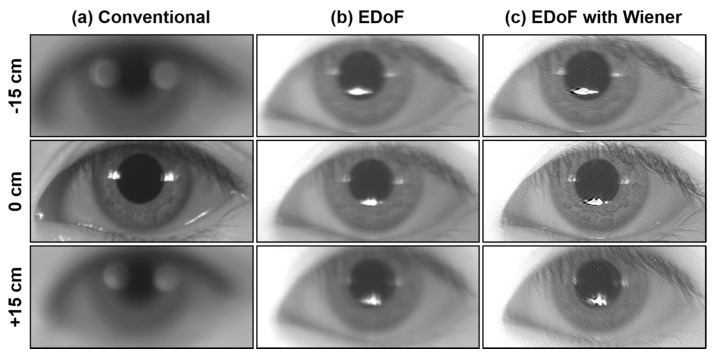
Iris image (640 × 480p) captured with different scenarios. From left to right: (**a**) conventional; (**b**) EDoF; and (**c**) EDoF with Wiener filtering. From top to bottom, the object distances are set to: (1) −15 cm; (2) on focus; and (3) +15 cm, respectively.

Figure 7 shows a series of iris images captured with respect to different object distances. Compared with the conventional image (left column), wavefront coding with (middle column) or without (right column) Wiener filtering effectively kept the iris image insensitive to the defocus effect. From the right column of Figure 7, EDoF with Wiener filter restoration manifested the detailed texture of iris image. However, the fidelity of the restored image was deteriorated by the artificial and ringing effect, respectively. The artificial effect was due to the wavefront error caused by the cubic phase mask. In an imaging optics, it is obvious that slight rotation and displacement of the phase components in a real system would induce the changes of phase error with respect to the focus [30].

**Figure 8:**
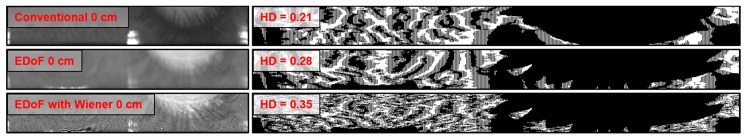
The intermediate images in stage of iris normalization and feature extraction (of images shown in Figure 7). The HD increased when wavefront coded image was used. The HD further increased when the wavefront coded image is restored using Wiener filtering.

The second factor is the ringing effect caused by Wiener filtering itself. As a phenomenon already presented in the literature [31,32], when images were restored by either the inverse linear filtering or the Wiener filtering, there would be a certain amount of noticeable edge error. For the inverse linear filtering, coded transfer function occurring to be zeros at high frequency caused singularities. For the Wiener filtering, though the above problem was solved by replacing the singularities of the inverse filter at zeros, there also existed edge error, as discussed in [30]. An optimization process on parameter *R* of the Wiener filtering may adequately reduce the edge error to some degree, but it is virtually impossible to remove all of it. In addition, even when the *R* value has been fine-tuned, the Wiener filtering could still cause a smearing effect near the center of the restored frequency spectrum, resulting in a reduction of the image’s resolution. Such a problem can be lessened or solved by further modification of the Wiener filtering, for example, using the method proposed in [32]. However, in order to restrict ourselves to focus on the main topic of this paper, we did not perform further analysis in this research direction. Detailed features were further examined on normalized iris image and iris code, as shown in Figure 8. The dissimilar iris code after Wiener filtering (bottom row) revealed that the restoration process was vulnerable to artificial noise and leads to increasing HDs.

### 4.3. Database

We collected iris images from 64 subjects (32 persons × 2 eyes) in National Chiao Tung University, Taiwan. Each subject stood at the on-focus position eight times, where the on-focus raw images and wavefront coded images were used as the different enrollment data. The total number of enrollment images was 512. Each subject stood at 11 defocus positions (from −15 to +15 cm, at 3-cm intervals) and was captured by both conventional and wavefront coded system. The total number of both conventional and wavefront coded probe images was 2816. The iris images were manually segmented and iris masks were also manually created. We used Libor Masek’s iris recognition toolbox written in Matlab for iris feature extraction [33], which used 1D Log-Gabor filters for iris feature extraction. After the iris codes were extracted, we computed normalized HD as described in Section 1. Each testing iris code was compared with the enrollment data. After all the possible combination comparison finished, we plotted the HD distributions for evaluation.

### 4.4. EDoF Performance Evaluation Method

Because we aimed to extend the DoF without compromising the iris recognition performance, the extension factor of DoF was defined as the longest object distance that can be achieved under the same error rate (i.e., accuracy invariance). Four error rates were used to examine the recognition performance: (1) false acceptance rate (FAR): the probability of falsely accepting an impostor as an authentic sample; (2) false rejection rate (FRR): the probability of falsely rejecting an authentic sample as an impostor sample; (3) equal error rate (EER): the value when FAR is equivalent to FRR; and (4) sensitivity index (SI): a measure to describe the separability between scores of authentic and impostor distributions. Sensitivity index is represented as follows.

(15) $$\text{SI}=\left(m_{im.}^{2}-m_{au.}^{2}\right)/\sqrt{\left(\sigma_{im.}^{2}-\sigma_{au.}^{2}\right)/2},$$

where m and σ are the mean and standard deviation, respectively. Afterward, we chose EER as the evaluation metric with different enrollment/testing schemes because EER is less sensitive to outliers.

### 4.5. Different Enrollment/Testing Schemes

The conventional scheme of an iris recognition system is to use the clear (on-focus) iris raw image as the enrollment data. During the testing stage, wavefront coded iris images were used as testing data. Such a scheme can be called heterogeneous matching. In this study, we would like to design a series of enrollment/testing pairs to test the feasibility of the combination of homogeneous and heterogeneous matching. A total of six approaches were carried out to inspect two issues: (1) whether the DoF of the iris recognition system can be effectively increased by employing the wavefront coded images as the enrollment data; and (2) among the various approaches, which (homogeneous or heterogeneous matching) approach is the best to balance the recognition accuracy and extended the DoF.

#### 4.5.1. Approach 1: Raw/Raw Pair

**Figure 9:**
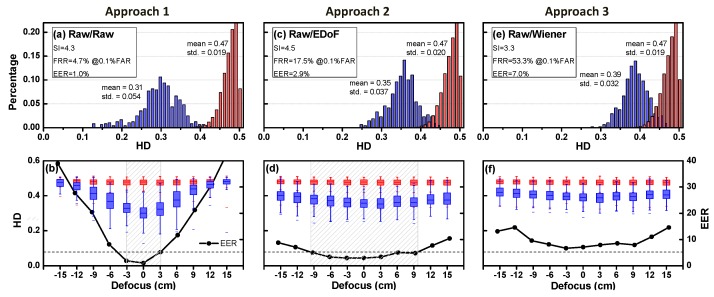
Experimental results, where the iris database (gallery images) is enrolled by the conventional optics, with testing images (probe images) captured by different schemes. **Top** row: HD Histogram distribution at on-focus: (**a**) conventional (Approach 1); (**c**) EDoF (Approach 2); and (**e**) EDoF with Wiener filtering (Approach 3). **Bottom** row: HD distribution (shown as the multiple boxplots colored in **red** and **blue**) and EER (shown as the **black** dotted curve) with different defocus: (**b**) Approach 1; (**d**) Approach 2; and (**f**) Approach 3. When EER was set to 5.2% as the baseline, Approach 2 extends the depth of field about 3.07-fold, whereas no improvement by Wiener filtering (Approach 3).

Approach 1 is the conventional imaging, where both enrollment and testing are not coded. The gallery images (i.e., enrollment) were iris images captured at on-focus position, while the probe images (i.e., testing) were iris images captured with various object distances. It can be considered a practice of “homogeneous matching”. Figure 9a shows the HD distribution when the subject stood on focus, where SI was 4.3, FRR was 4.7% when FAR was 0.1%, and EER was 1.0%. Blue and red bars represent the distribution of the HD of the authentic and impostor matching results, respectively. Such results were reasonable based on the small number of test subjects. For example, in the result of the Multiple Biometric Grand Challenge (MBGC) 2009 version 2, it reports that the best four groups had FRR ranging from roughly 10% when FAR was set to 0.1% [34]. Such great results were computed based on a dataset which consists of 4789 right iris and 4792 left iris images of 136 subjects. With much less iris data, our recognition rate outperformed theirs. In this way, the quality of our iris recognizer was assured, since our recognition results were comparable to the best four groups from the MBGC 2009 version 2.

Figure 9b shows HD versus defocus. The boxplot represents the first quartile to third quartile of the data, while the five error bars from the top to the bottom represent the maximum, 99%, median, 1 percent and minimum values of the data. The HD of authentic matching rapidly increased as the subject was out of DoF, whereas the impostor matching was kept at a high value. The increasing authentic HD was due to the quality heterogeneity of iris images with defocus effect. The trend of EER was in close agreement with the theoretical prediction in Section 2.2. If we set ±30-mm as the DoF, the EER = 5.2% was defined as the baseline for the following comparison.

#### 4.5.2. Approach 2: Raw/EDoF Pair

Approach 2 was the case where the wavefront coded images were captured as probe images. The gallery images were the same as Approach 1. It can be considered = a practice of “heterogeneous matching”. Figure 9c shows the authentic and impostor HD histogram when the subjects were on focus. The SI was 4.5, FRR was 17.5% when FAR was 0.1%, and EER was 2.9%. As expected, the performance of recognition at the best focus was poorer than the conventional one due to a prior phase modulation.

Figure 9d shows the authentic and imposter HD with defocus. Compared to conventional imaging (Approach 1), EERs were increasing less rapidly with respect to increasing defocus. With the same merit in terms of EER was 5.2%, the DoF by wavefront coding was extended by a factor of 3.07. Such results validated the feasibility of DoF theory in Section 2.2, where the extended factor could be higher if the lower F-number optics were used. 

#### 4.5.3. Approach 3: Raw/Wiener Pair

In this approach, we aimed to examine the performance of restoration of coded iris images (probe images). The gallery images were the same as Approach 1, and the probe images were coded iris images after Wiener filtering. It can be considered another practice of “heterogeneous matching”. Figure 9e shows the authentic and impostor HD histograms. The SI was reduced to 3.3, FRR was 53.3% when FAR was 0.1%, and EER was 7.0%. The large amount of overlapping would prevent the practical use of the system in most recognition requests. 

Figure 9f shows that high EER over the capture zone causing the system to be highly unstable. Some of the literature claimed that a perfect digital filter had the capacity to restore the coded iris image over an extended DoF without adversely affecting the recognition accuracy. Unfortunately, in this study, no improvement was observed compared to the conventional iris imaging system (Approach 1) as well as wavefront coding image without the restoration (Approach 2).

#### 4.5.4. Approach 4: EDoF/EDoF Pair

Iris recognition relies heavily on the correct feature matching of the iris codes between enrollment and test images. The failure of Approach 3 inspired us to investigate the performance of recognition with a new EDoF enrollment. In this approach, both the gallery images and the probe images were iris images acquired by a wavefront coded image without any restoration process. The difference between them lies in that gallery images were captured in focus, while the probe images were captured at various defocus positions. It can be considered another practice of “homogeneous matching”.

**Figure 10:**
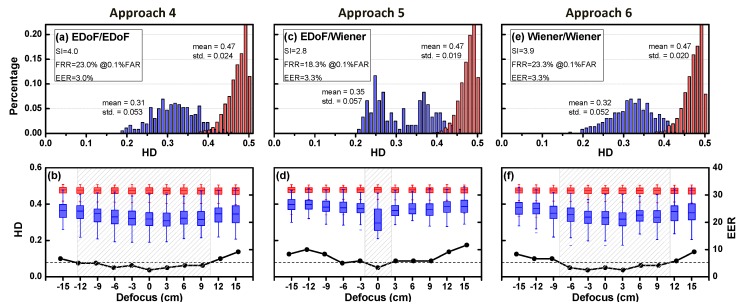
Experimental results, where the iris database (gallery images) is enrolled by the EDoF image (Approach 4 and 5); and EDoF+Wiener image (Approach 6), respectively; For different enrollment/testing pairs, the homogeneous imaging pairs (Approaches 4 and 6) is superior to heterogeneous one (Approach 5) in terms of recognition rate. **Top** row: HD Histogram distribution at on-focus: (**a**) EDoF/EDoF (Approach 4); (**c**) EDoF/Wiener (Approach 5); and (**e**) Wiener/Wiener (Approach 6). **Bottom** row: HD distribution (shown as the multiple boxplots colored in **red** and **blue**) and EER (shown as the **black** dotted curve) with different defocus: (**b**) Approach 4; (**d**) Approach 5; and (**f**) Approach 6. Compared to the conventional optics with EER was set to 5.2%, the DoF was extended by a factor of 3.71 (Approach 4) and 3.10 (Approach 6), respectively.

Figure 10a shows the authentic and impostor HD histogram on focus. The SI was 4.0, FRR was 23.0% when FAR was 0.1%, and EER was 3.0%. Figure 10b shows authentic and impostor HD versus defocus range. With the same baseline (EER = 5.2%), the DoF was extended by a factor of 3.71. Surprisingly, the extended factor was higher than Approach 2. Compared with different types of the gallery and the probe images (i.e., heterogeneous pair) in Approach 2, the recognition rate was improved by the same types of gallery and the probe images (i.e., homogeneous pair).

#### 4.5.5. Approach 5: EDoF/Wiener Pair

For the sake of completeness of this study, we also examined the recognition performance through the Wiener filtering with EDoF enrollment. In this approach, the gallery images were the same as in Approach 4, while the probe images were coded iris images with restoration by Wiener filtering. It can be considered as another practice of “heterogeneous matching”. Figure 10c shows the authentic and impostor HD histogram. The SI was 2.8, FRR was 18.3% when FAR was 0.1%, and EER was 3.3%. High EERs showed that the iris codes were dramatically changed by the noticeable artifacts from Wiener filtering. Therefore, Approach 5 was not suggested for the purpose of iris recognition.

#### 4.5.6. Approach 6: Wiener/Wiener Pair

In the last scheme, both the gallery and the probe images were coded iris images restored by Wiener filtering. It can be considered another practice of “homogeneous matching”. We aimed to check whether the homogeneous acquisition scheme (EDoF with Wiener filtering) in both enrollment and testing would alleviate the side effects caused by the Wiener filtering. Figure 10e shows the authentic and impostor HD histogram. The SI was 3.9, FRR was 23.3% when FAR was 0.1%, and EER was 3.3%. Figure 10f shows authentic and impostor HD versus defocus range.

For wavefront coded image, the performance of homogeneous pairs (Approaches 4 and 6) were better that of heterogeneous pairs (Approach 5). Compared to Approach 4, the global distribution of the HD in Approach 6 showed a decreasing trend, as could be observed for both authentic and imposter HD distributions. Such property reveals that after performing image restoration using the Wiener filtering, the induced artifacts would make images of different classes look more similar to each other, causing a decreased HD for inter-class comparison. For the purpose of biometric identification, such a phenomenon was not desirable and should be avoided.

## 5. Discussion

In this paper, six enrollment/testing system configurations were carried out for the iris recognition system. These configurations can be divided into homogeneous pairs (Approaches 1, 4 and 6) and heterogeneous pairs (Approaches 2, 3 and 5). Based on the statistical results summarized in Table 2, the DoF of the wavefront coding image was significantly extended. Taking the best scheme (Approach 4), the factor was 3.71 based on the criteria EER = 5.2%. For optimal system in operation, we suggest using the homogeneous optics (Approach 1, 4 or 6) to achieve a more satisfactory recognition rate. However, if we consider the power of the EDoF capability as one of the core objective functions from the experimental results, Approach 4 is the best approach with a 3.71 EDoF factor.

One lesson we learned from the experiment is how to design an imaging system for the purpose of pattern recognition. In order to achieve the highest recognition rate, one should make sure to put into the gallery set those images which were processed in exactly the same procedure as the test images. Otherwise, the heterogeneity caused by the hardware mismatch would degrade the accuracy. From the perspective of pattern recognition theory, it is better that the gallery image set involves the largest possible amount of variations which could possibly be observed in the probe image set. In such conditions, pattern recognition or machine learning algorithms could estimate the density of the image sample distribution correctly. During the testing stage the learned decision boundary can be robust enough to achieve higher recognition rate.

Another question we can ask ourselves is, given the aforementioned principle, why is the performance of Approach 4 better than 6? As discussed in Section 4.2, using Wiener filtering for image restoration may introduce additional artifacts, which may degrade the image and modify the detailed textural structures in iris images. Iris recognition relies heavily on the correct pattern matching of the iris code between training and test images. If the detailed textural components of an iris image are changed by some unpredictable factor, the iris code changes dramatically. That is the reason why the recognition performance of Approach 6 is worse than 4. Such experimental results also coincide with the practice proposed in [11], which shows that such methodology is supported by two independent research groups.

The comparison between the proposed method in the best scheme and existing works is summarized in Table 3 [7,8,9,10,11]. Compared to other works, the desired distance is set to 300 cm for a long range iris recognition system. As it is strict and harsh enough to test the practicality and feasibility of the EDoF-empowered image sensors, the database is abundantly captured. Due to the difficulty of long range image acquisition, the optics, sensor and wavefront coding technique are systematically designed and integrated into our laboratory. Finally, the EDoF factor reached 3.71 times that of the original system without loss of recognition accuracy.

## 6. Conclusions

In this paper, we examine a number of EDoF approaches for the purpose of a distant iris recognition system. Unlike prior studies that mostly addressed this in a simulation, we experimentally overhauled the entire computational imaging flow via an EDoF imagery and verified the ultimate performance with different homo- and hetero-enrollment/testing image pairs. 

On the basis of experimental results, the DoF of the wavefront coding system is significantly increased in comparison with the conventional imaging. Taking the best scheme (Approach 4) as the benchmark, the EDoF factor was 3.71 under the constraint EER = 5.2%. For optimal system configurations of testing and enrollment image sets, we suggest using the homogeneous pair (Approaches 1, 4 and 6) to achieve a more satisfactory recognition rate. 

The EDoF function via pupil engineering is validated based on the assumption that the pupil mask should be in place of the pupil or equivalent in the imaging system. For practical use, different positions of the phase mask would lead to diverse coding effects with respect to field of view. As a result, the fidelity of the restored image is difficult to keep constant within a wide acquisition volume. To keep the uniform phase coding satisfying the linear shift invariant, the position of the aperture stop in the system layout should be further examined in future work.

## Figures and Tables

**Table 1:** Specification of image sensor and telephoto lens set.

MV1-D2080 IR Sensor		Sigma APO 150–500 mm	
Optical Format	23.5 mm	Field of View	5–16 degrees
Resolution	2080 × 2080	Minimum Distance	220 cm
Pixel Size	8 μm	Maximum Mag.	1:5.2
Dark current	0.65 fA/pixel	Caliber Diameter	86 mm

**Table 2:** The results of six experimental approaches.

Test Enrollment	Conventional	EDoF	EdoF with Wiener
Conventional	DoF = 6 cm	DoF = 18.4 cm	DoF = 0 cm
	SI = 4.3	SI = 4.5	SI = 3.3
	FRR = 4.7%	FRR = 17.5%	FRR = 53.3%
	EER = 1.0%	EER = 2.9%	EER = 7.0%
EdoF		DoF = 22.2 cm	DoF = 4.5 cm
		SI = 4.0	SI = 2.8
		FRR = 23.0%	FRR = 18.3%
		EER = 3.3%	EER = 3.3%
EdoF with Wiener			DoF = 18.6 cm
			SI = 3.9
			FRR = 23.3%
			EER = 3.3%

**Table 3:** Comparison between existing works and this study.

	Proposal	Gracht [7]	Narayanswamy [8]	Smith [9]	Barwick [10]	Boddeti [11]
Scheme	Experiment	Experiment	Simulation	Simulation	Simulation	Simulation
Database	Laboratory	Laboratory	Laboratory	ICE	UPOL	ICE
	3328 images 64 classes	-	44 images	150 images	168 images	1061 images
		one class	two classes	50 classes	56 classes	61 classes
Distance	300 cm	50 cm	55 cm	50 cm	55 cm	-
Optics	f = 400 mm	f = 57 mm	f = 50 mm	f = 53 mm	f = 50 mm	-
	F/6.3	F/8	F/3.5	F/2	F/2.85	
	λ = 850 nm	λ = 830 nm	λ = 780 nm	λ = 760 nm	λ = 768 nm	
Sensor	2080 × 2080	1300 × 1300	1024 × 768	-	-	-
	8 μm	6.7 μm	-	5.134 μm	3 μm	
Wavefront coding	Cubic	Cubic	Cubic	Cubic	Cubic-pentic	Cubic
	α = 42	α = 11	α = 156	α =30	(−16, 71, −265, 370, 267)	α = 60
Restoration	without	with	with	without	without	without
Merit function	Accuracy invariant	HD = 0.32	Iris score ^1^ set to 0.3	HD = 0.33	SI = 5	Error bars of the authentic and impostor scores do not overlap
Extended factor	3.71	over 2	over 3.3	2.8	2.2	4.8

^1^ Iris score: using exclusive-NOR operator for bit comparison. The values 1 and 0 represents the match and mismatch bit pairs, respectively.
